# Effect of *Ampelopsis brevipedunculata* (Maxim.) Trautv extract on a model of atopic dermatitis in HaCaT cells and mice

**DOI:** 10.1002/fsn3.3610

**Published:** 2023-08-17

**Authors:** Seon Gyeong Bak, Hyung Jin Lim, Yeong‐Seon Won, Eun Jae Park, Young Hee Kim, Seung Woong Lee, Je Hun Oh, Ji Eun Kim, Min Jee Lee, Soyoung Lee, Seung Jae Lee, Mun Chual Rho

**Affiliations:** ^1^ Functional Biomaterial Research Center Korea Research Institute of Bioscience and Biotechnology (KRIBB) Jeongeup South Korea; ^2^ Division of Biotechnology and Advanced Institute of Environment and Bioscience College of Environmental and Bioresource Sciences, Jeonbuk National University Iksan South Korea; ^3^ Ju Yeong NS Co., Ltd Seoul South Korea; ^4^ Applied Biological Engineering, KRIBB School of Biotechnology University of Science and Technology Daejeon South Korea

**Keywords:** *Ampelopsis brevipedunculata*, atopic dermatitis, skin barrier, skin inflammation

## Abstract

*Ampelopsis brevipedunculata* (Maxim.) Trautv. has been used for a long time as a folk remedy. According to studies, it possesses anti‐inflammatory, antioxidant, and antibacterial properties. However, its effects on atopic dermatitis (AD) are poorly studied. Thus, we investigated the therapeutic effect of *A. brevipedunculata* (Maxim.) Trautv. extract (ABE‐M) on 2,4‐dinitrochlorobenzene (DNCB)‐induced AD. For in vitro analysis, keratinocytes cell lines (HaCaT cells) were used. To evaluate the gene and protein expression levels of cytokines and chemokines, TNF‐α/IFN‐γ‐stimulated HaCaT cells were treated with ABE‐M. The cells and the supernatant were collected, then gene and protein levels were analyzed by real‐time polymerase chain reaction and enzyme‐linked immunosorbent assay analysis. For in vivo analysis, BALB/c mice (6 weeks) were randomly separated into five groups (*n* = 5). The mice were applied DNCB and phosphate‐buffered saline, dexamethasone (DX) or ABE‐M (50, 100, and 200 mg/kg) was orally administrated for 28 days. At the end, ear tissues and blood were collected for histological analysis and evaluation of cytokines and chemokines. In keratinocytes, ABE‐M inhibited the protein and mRNA levels of chemokines, and cytokines exposed by TNF‐α/IFN‐γ. Similarly, the expression of chemokines was suppressed by ABE‐M in AD animal model induced by DNCB and the level of pro‐inflammatory cytokines was decreased in a dose‐dependent manner. Our research indicates that ABE‐M could be a candidate material that can be used to improve skin immunity enhancement, health, and beauty.

## Graphical abstract

This study researched the effects of *Ampelopsis brevipedunculata* (Maxim.) Trautv. extract (ABE‐M) on HaCaT cells and 2,4‐dinitrochlorobenzene‐induced atopy diseases, and the findings indicated that ABE‐M may be useful as a functional food and therapeutic agent.

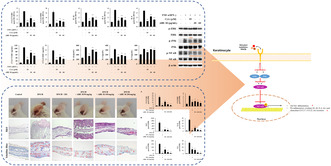

## 1 INTRODUCTION

The skin is at the forefront through which the body limits moisture loss and prevents the ingress of detrimental environmental substances and microorganisms (Nestle et al., 2009). The skin contains epithelium, stroma, and immune cells, which interact to influence local and systemic immunity (Di et al., 2011). Keratinocytes are mainly involved in the immunity of the skin, which is colonized by non‐epithelial immune cells (dendritic epithelial T cells and Langerhans cells) (Pasparakis et al., 2014; Walters & Roberts, 2002). Immunomodulation of these cells contributes to disease onset and exacerbation in atopic dermatitis (AD) (Di et al., 2011). AD is a kind of chronic skin inflammatory disease, recognized as Th2 responses (Brandt & Sivaprasad, 2011). The predominant Th2‐type cytokines are deeply involved in the initiation and exacerbation of AD (Brandt & Sivaprasad, 2011; Raap et al., 2012; Ren et al., 2022). Since mediating AD can prevent the development of skin inflammation, timely treatment is important for skin health. Thymus and activation‐regulated chemokine (CCL17/TARC) and macrophage‐derived chemokines (CCL22/MDC) were detected in inflamed skin of patients with AD (Homey et al., 2006; Nakazato et al., 2008). CCL17/TARC is expressed during the pathogenesis of allergic diseases such as AD and is selectively expressed in Th2 cells (Furukawa, Nakamura, et al., 2004; Saeki & Tamaki, 2006). CCL17/TARC and CCL22/MDC increase infiltration of CC chemokine receptor 4 (CCR4) expressing cells, such as Th2 lymphocytes, basophils, and natural killer cells, by binding to CCR4 (Saeki & Tamaki, 2008). They also promote the differentiation of Th2 cells (Hirata et al., 2019). In addition, CCL22/MDC has been reported to be interrelated with rash strength in children with AD (Hashimoto et al., 2006). This finding suggests that CCL17/TARC and CCL22/MDC would be good markers for AD.

*Ampelopsis brevipedunculata* (Maxim.) Trautv. has been used as a folk medicine since ancient times. It has been reported in previous studies that the vine of *A. brevipedunculata* (Maxim.) Trautv. has excellent effects in the treatment of liver disease and liver fibrosis (Yum et al., 2017). Moreover, its antioxidants (Kundaković et al., 2008), antibacterial activities (Kundakovic et al., 2008), and its effects against Alzheimer's disease have been reported in previous studies (Rashed et al., 2015). Recently, *A. brevipedunculata* (Maxim.) Trautv. ethanol extract has been reported to inhibit the effect of STAT3 activity on IL‐6 in inflammatory and immune diseases (Jang et al., 2018). In addition, studies have shown that this agent is effective for treating DFE/2,4‐dinitrochlorobenzene (DNCB)‐induced AD such as skin inflammation (Choi et al., 2019).

In this study, we studied the effects of *A. brevipedunculata* (Maxim.) Trautv. extract (ABE‐M) on HaCaT cells and DNCB‐induced AD and the findings indicated that ABE‐M may be useful as a functional food and therapeutic agent for AD.

## 2 MATERIALS AND METHODS

### 2.1 Preparation of ABE‐M

*Ampelopsis brevipedunculata* (Maxim.) Trautv was obtained from HeukSaRang agricultural association corporation (Yeongju, Korea). The authenticity of the plant was verified by DowGene Co., Ltd (Seoul, Korea) using DNA analysis. Dried the vine of *A. brevipedunculata* (Maxim.) Trautv was extracted with 55% alcohol (468 L) at 78°C (9 h) and filtered using a 1 μm cartridge filter. Then, the filtrates were concentrated to 20% solid content. An appropriate amount of excipient (maltodextrin, Daesang Co., Ltd.) was added, mixed, and spray‐dried, resulting in a component ratio of 80% of ABE and 20% of the excipient. Also, 4.8 kg of ABE preparation in total produced a yield of 12.3% (ABE‐M, Production No. JY206MM210930).

### 2.2 Preparation of ABE‐M for HPLC and HPLC‐UV analysis

ABE‐M (1.0 g) was relocated to a 100 mL volumetric flask and was dissolved 50% MeOH (100 mL) to obtain the 10 mg/mL concentration. Then, 1 mL of solution was filtered using a hydrophobic syringe filter unit (Thermo scientific, 13 mm, 0.45 μm). The chromatographic peaks of the sample solution were confirmed by comparing the retention time with the standard material. Quantitative analysis was done by the integration of peaks through an external standard method.

The reference standards of catechin and ethyl gallate were purchased from Sigma‐Aldrich, Inc. (St. Louis, MO, USA). The standard stock solutions of two reference standards were dissolved in 50% MeOH to obtain 10 mg/mL concentration. The working solutions were prepared using serial dilution of stock solutions to seven concentrations, 2.0–250.0 μg/mL with 50% MeOH. Linear regression data were determined by calculating the integrated peak area (*y*) of each concentration (*x*, μg/mL) versus the continuous minimum concentration. Two reference standards were expressed as milligrams per gram of extract. HPLC‐UV analysis was done on an Agilent 1200 series HPLC instrument armed with a vacuum degasser, a binary pump, a VWD detector, and a temperature‐controlled column oven. The calibration standards and sample extracts were injected via a 10 μL sample loop and the Phenomenex C18 column (250 × 4.6 mm, 5 μm) was adjusted for the analysis. The detection wavelength was 275 nm and the column temperature were set at the 25°C. The mobile phase consisted of 0.1% trifluoroacetic acid solution (solvent A) and acetonitrile (solvent B). The gradient mode was as follows: 10% B for 0–5 min; 10%–13% B for 5–7 min; 13% B for 7–15 min; 13%–20% B for 15–35 min; 20%–100% B for 35–36 min; and 100% B for 36–50 min. The flow rate was set at 1.0 mL/min. HPLC chromatogram is shown in Figure S1 and the amount of catechin and ethyl gallate in 1 g of ABE‐M was 17.73 ± 0.81 and 2.97 ± 0.08 mg.

### 2.3 Cell culture

The human keratinocyte cell line (HaCaT cells) was subcultured according to the laboratory method at 37°C with 90%–95% humidity and 5% CO_2_. The composition of the culture medium is as follows: Dulbecco's Modified Eagle's Medium, supplemented with 10% heat‐inactivated fetal bovine serum, and 1% penicillin–streptomycin.

### 2.4 Cell viability assay

Cell viability was determined by the 3‐(4,5‐dimethylthiazol‐2‐yl)‐2,5‐diphenyl tetrazolium bromide (MTT) assay, which was performed as the same method in previous studies (Bak et al., 2022). Different concentrations (10, 30, 60, and 100 μg/mL) of ABE‐M were used.

### 2.5 Enzyme‐linked immunosorbent assay

HaCaT cells were seeded 1 × 10^6^ cells/well in 6‐well plates. After incubating overnight, they were pretreated with ABE‐M or cyclosporine A for 1 h. Then, after being stimulated with TNF‐α (50 ng/mL)/IFN‐γ (50 ng/mL), the cells were incubated for 24 h. Cyclosporin A was used as positive control, following a previous study (Choi et al., 2013). Cytokines (IL‐1β, IL‐6, and IL‐8) and chemokines (CCL17/TARC, CCL22/MDC) levels in the cell culture medium were measured using an enzyme‐linked immunosorbent assay (ELISA) kit (BD Biosciences, San Diego, CA, USA, R&D systems, Minneapolis, MN, respectively), and Serum Immunoglobulin (Ig) E, G1, and G2a from mouse blood were determined using an ELISA kit (BD Biosciences) according to the manufacturer's instructions. The protein (p‐ERK, p‐JNK and p‐NF‐κB) from mouse ear tissue were determined using an ELISA kits (Cell Signaling Technology). The absorbance was measured at 450 nm using a microplate reader.

### 2.6 Real‐time polymerase chain reaction

Total RNA from HaCaT cells and mouse ear tissue was isolated using TRIzol reagent. Real‐time polymerase chain reaction (PCR) was performed as the same method in the previous study (Bak et al., 2022), and as endogenous controls for normalization, eukaryotic 18s rRNA and mouse GAPDH was used. TaqMan primers and probes (Applied Biosystems, Thermo Fisher Scientific) were used as follows: interleukin (IL)‐1β (Hs01555410_m1, Mm00434228_m1), IL‐4 (Mm00445259_m1), IL‐6 (Hs00174131_m1, Mm00446190_m1), IL‐8 (Hs00174103_m1), CCL17 (Hs00171074_m1, Mm01244826_g1), CCL22 (Hs01574247_m1, Mm00436439_m1), and TSLP (Mm01157588_m1).

### 2.7 Western blotting

Detailed protein extraction and western blot procedures applied here are described in previous studies (Bak et al., 2022, 2023). Total protein was isolated in 100 μL of cell lysis buffer (Cell Signaling Technology, Danvers, MA, USA) from HaCaT cells stimulated with TNF‐α (50 ng/mL)/IFN‐γ (50 ng/mL) for 30 min. Quantification of proteins was performed with the DC Protein Assay Kit (Bio‐Rad). Identical quantified protein lysates were electrophoresed on SDS‐PAGE gels and then transferred to polyvinylidene fluoride (PVDF) membranes. Primary antibodies against target proteins (p‐ERK, p‐JNK, p‐NF‐κB and b‐actin) were identified. Bands were developed using the West‐Queen RTS Western Blot Detection Kit (iNtRON Bio). Antibodies purchased from Cell Signaling Technology and Santa Cruz were used.

### 2.8 Animal model

BALB/c mice (female, 6 weeks) were purchased from Samtako (Osan, Korea). All animals were housed during the study period in the same environment as presented in previous studies (Bak et al., 2022). Animal care and treatment protocols were conducted according to the guidelines established by the Public Health Service Policy on the Humane Care and Use of Laboratory Animals and were approved by the Institutional Animal Care and Use Committee of the Korea Research Institute of Bioscience and Biotechnology (KRIBB‐AEC‐21259, October 25, 2022).

### 2.9 Generation of DNCB‐induced AD‐like skin lesions

A total of 25 mice (*n* = 5/group) were divided into the phosphate‐buffered saline (PBS) vehicle, DNCB vehicle (PBS), DNCB plus ABE‐M (50, 100, and 200 mg/kg), and DNCB plus DX (1 mg/kg) groups. In the first week of induction, the mice were sensitized by the application of DNCB (2%, 20 μL/ear) once on each ear. After 1 week, both ears of each BALB/c mouse were challenged with DNCB (1%, 20 μL/ear, twice/week) for 4 weeks. During the DNCB challenge, ABE‐M or DX was administered orally by gavage for five consecutive days per week. DX was used as positive control, based on previous studies (Choi et al., 2013; Jeong et al., 2020). After the experiment, the mice were sacrificed by cervical dislocation under isoflurane anesthesia.

### 2.10 Histological analysis

Mouse ear tissue was fixed with 10% (w/v) para‐formaldehyde. Paraffin‐embedded tissues were sectioned at 3 μm thickness by microtome. After removing paraffin, they were stained with hematoxylin and eosin (H&E) and toluidine blue.

### 2.11 Statistical analysis

Statistical analysis was performed using Prism 5 software (GraphPad Software). The data are presented as the mean ± SD of nine individual experiments. Statistical significance was determined by one‐way ANOVA followed by Tukey's multiple comparisons tests.

## 3 RESULTS

### 3.1 Effect of ABE‐M on the viability in HaCaT cells

**FIGURE 1:**
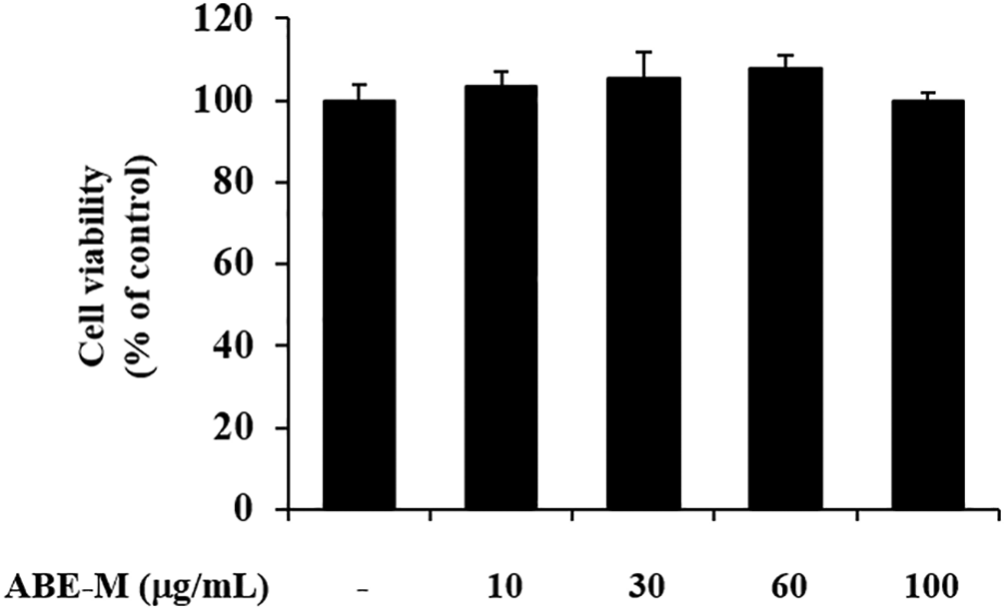
Effect of ABE‐M on cell viability. The viability of ABE‐M‐treated HaCaT cells was assessed by MTT assay. *p < .05, **p < .01 compared to the control. ABE‐M, *Ampelopsis brevipedunculata* (Maxim.) Trautv. extract; MTT, 3‐(4, 5‐dimethylthiazol‐2‐yl)‐2, 5‐diphenyl tetrazolium bromide.

Before investigating the anti‐inflammation activity of ABE‐M, the cell viability of ABE‐M treated HaCaT cells was evaluated using MTT assay. The results confirmed that ABE‐M was not cytotoxic at 10 to 100 μg/mL (Figure 1). Therefore, subsequent experiments proceeded using 30 and 60 μg/mL concentrations of ABE‐M.

### 3.2 Effect of ABE‐M on chemokine and cytokine levels in HaCaT cells

**FIGURE 2:**
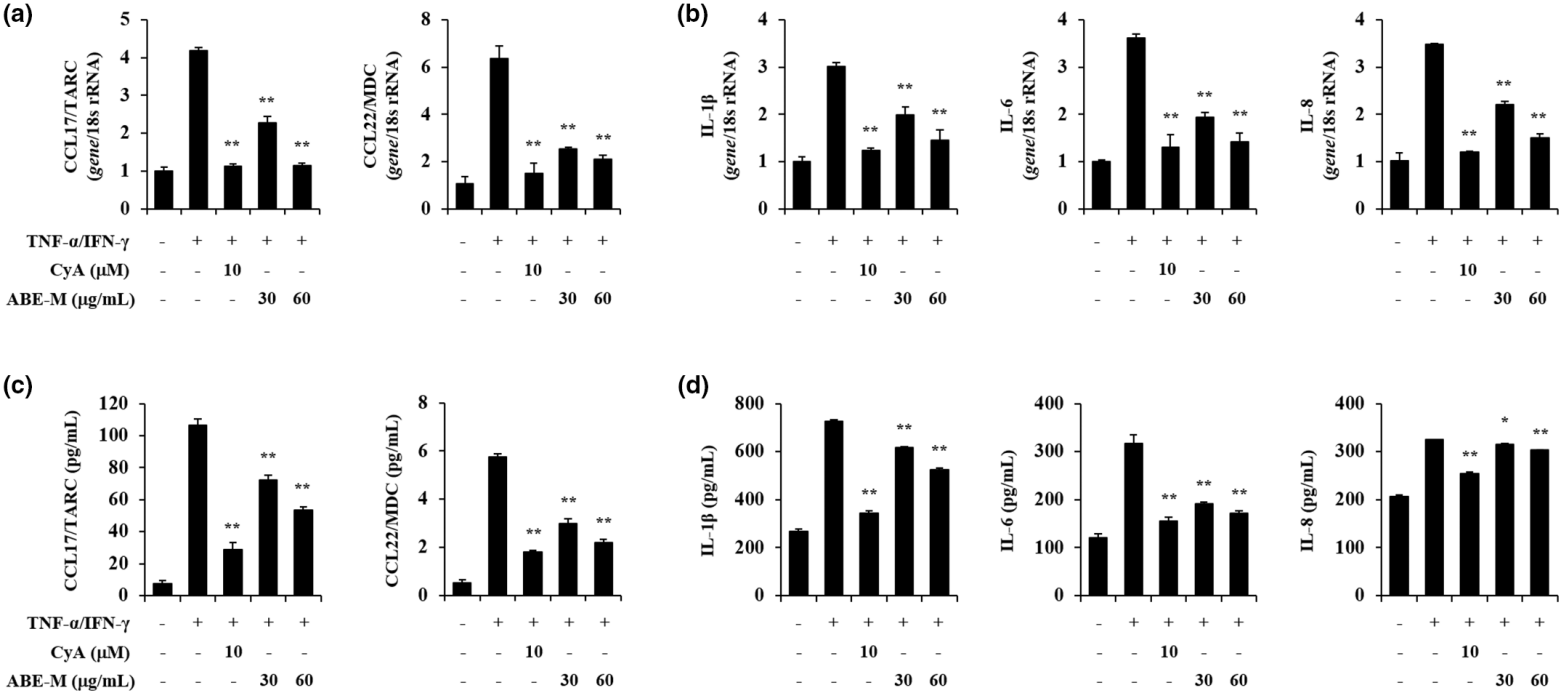
Effect of ABE‐M on pro‐inflammatory cytokine secretion in TNF‐α/IFN‐γ‐stimulated HaCaT cells. The gene expression of pro‐inflammatory chemokines (a) and cytokines (b) was detected by using real‐time PCR. The protein levels of pro‐inflammatory chemokines (c) and cytokines (d) in the culture supernatants were measured by ELISA. *p < .05, **p < .01 compared to the TNF‐α/IFN‐γ group. ABE‐M, *Ampelopsis brevipedunculata* (Maxim.) Trautv. extract; CyA, cyclosporine A; ELISA, enzyme‐linked immunosorbent assay; PCR, polymerase chain reaction.

The effect of ABE‐M was evaluated using real‐time PCR and ELISA. Human keratinocytes, HaCaT cells were stimulated with the pro‐inflammatory mediator TNF‐α/IFN‐γ. The expression of chemokines such as CCL17 and CCL22, which are biomarkers of AD, in the stimulated group was compared with the control group (Figure 2a). The expression of pro‐inflammatory cytokines, including IL‐1β, IL‐6, and IL‐8 (*p* < .05, *p* < .01) was elevated in the TNF‐α/IFN‐γ treatment group contrasted to the control group (Figure 2b). However, the increase in gene expression was abrogated by treatment with the ABE‐M in a concentration‐dependent manner (*p* < .05, *p* < .01; Figure 2a,b). Furthermore, 60 μg/mL of ABE‐M suppressed these genes similar to cyclosporine A. The protein levels of chemokines and cytokines showed the same trends as in gene expression (*p* < .05, *p* < .01) (Figure 2c,d). Therefore, our results indicated that the treatment with ABE‐M suppresses AD and inflammatory response mediators in keratinocytes.

### 3.3 Effect of ABE‐M on protein levels in HaCaT cells

**FIGURE 3:**
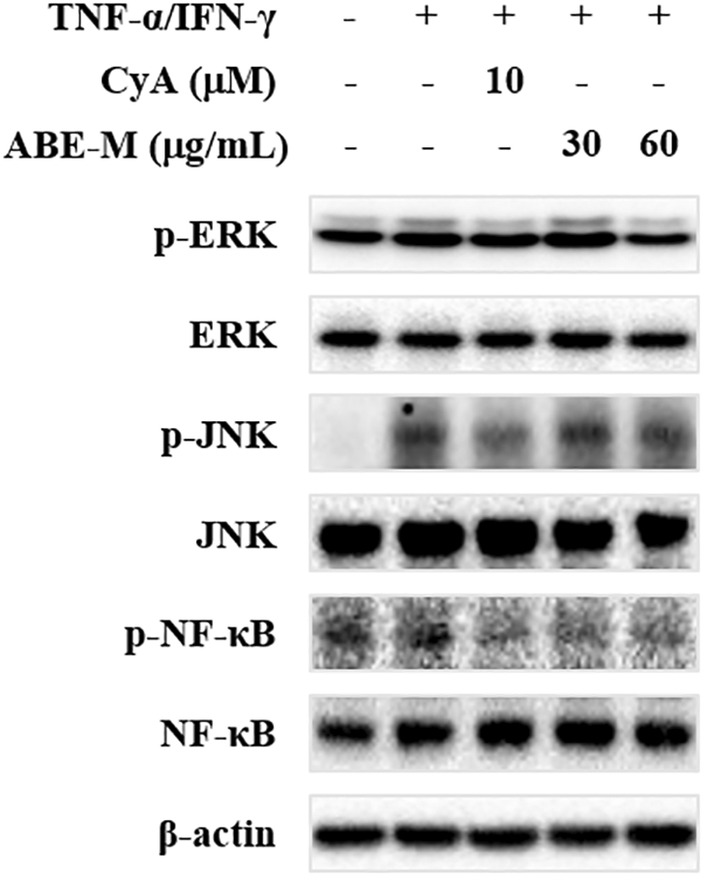
Effect of ABE‐M on HaCaT cell signaling pathway. The protein level was confirmed in the signaling pathway in HaCaT cells by western blot. ABE‐M, *Ampelopsis brevipedunculata* (Maxim.) Trautv. extract.

The effect of ABE‐M on signaling pathway was evaluated using western blot. HaCaT cells were stimulated with the TNF‐α/IFN‐γ for 30 min. The expression of p‐ERK and p‐JNK was elevated in the TNF‐α/IFN‐γ‐stimulated group compared to that of the control group (Figure 3). However, the elevated expression was reduced by the treatment of 60 μg/mL of ABE‐M. Moreover, it was confirmed that p‐NF‐κB was also inhibited by ABE‐M. Thus, the inhibition of these signaling pathways by ABE‐M indicated that ABE‐M inhibits the secretion of cytokines and chemokines through these pathways.

### 3.4 Effect of ABE‐M on AD‐like skin lesions in BALB/c mice

**FIGURE 4:**
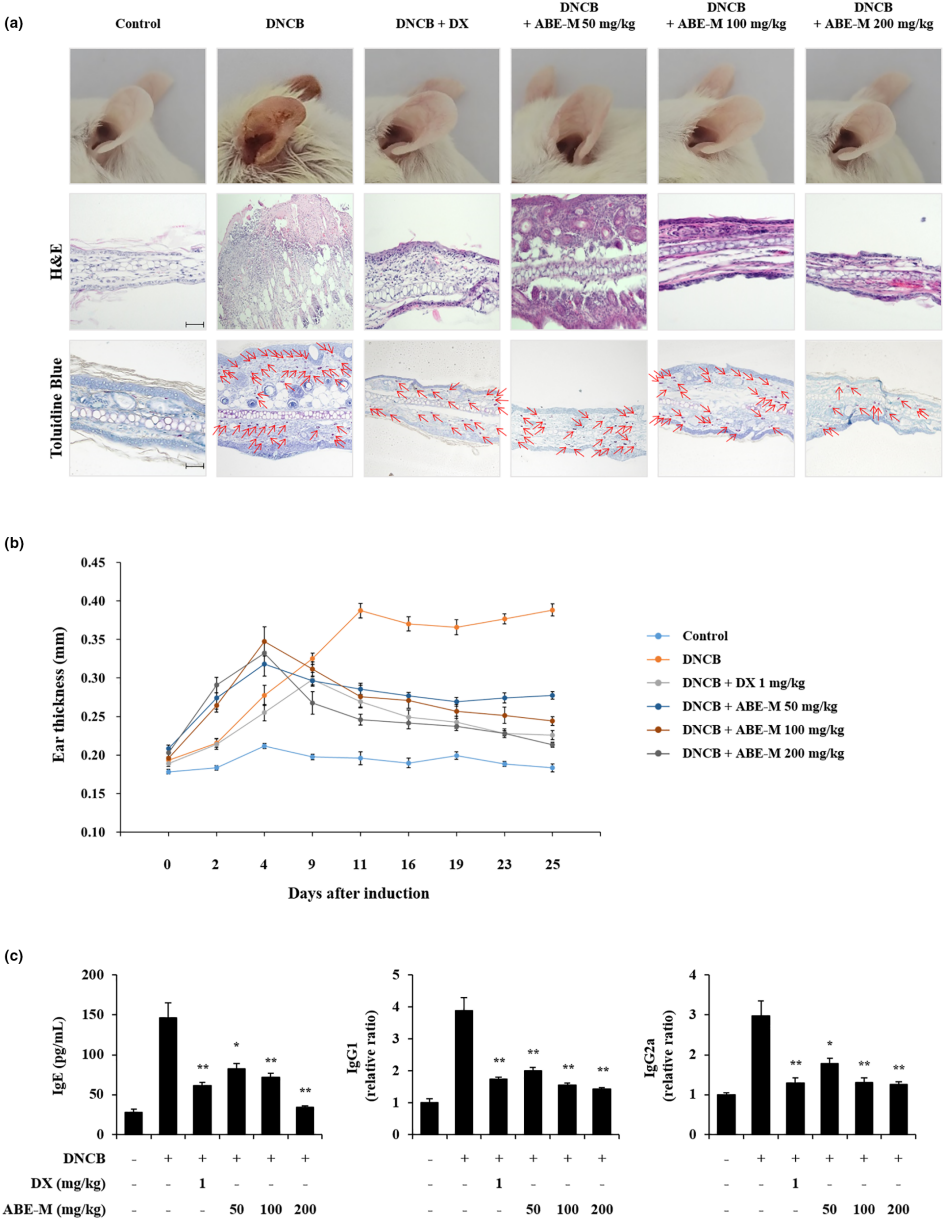
Effect of ABE‐M on skin lesions and IgE, IgG1, and IgG2a levels in DNCB‐induced AD mouse model. (a) The ear tissue was fixed with 10% formalin and paraffin‐embedded. The paraffin blocks were cut into 3 μm sections and stained with H&E and toluidine blue. Sections were evaluated at an original magnification of 200×, scale bar = 100 μm. (b) Ear thickness was assessed using a micrometer on days after DNCB induction (c) Serum IgE, IgG1, and IgG2a levels were determined by ELISA. *p < .05, **p < .01 compared to the DNCB group. ABE‐M, *Ampelopsis brevipedunculata* (Maxim.) Trautv. extract; AD, atopic dermatitis; DNCB, 2,4‐dinitrochlorobenzene; ELISA, enzyme‐linked immunosorbent assay; H&E, hematoxylin and eosin.

To evaluate the anti‐AD effect of ABE‐M, an animal model was constructed by applying DNCB in the ears of mice for 4 weeks. Reiterated topical skin application of DNCB twice per week significantly increased ear edema compared to that in the control group. However, the ABE‐M treated group showed a greater decrease in ear edema than the DNCB group (Figure 4a upper panel). To analyze the ABE‐M effect on skin hypertrophy, ear tissues were stained with H&E and observed by light microscopy. Continuous DNCB exposure caused strong inflammatory changes such as thickening of the dermis and epidermis in the ear tissues of AD mice compared to the control group (Figure 4a middle panel). It has been reported that mast cell infiltration occurs in skin lesions such as atopy. Therefore, we investigated the mast cell infiltration through toluidine blue staining. Mast cell infiltration was increased by DNCB compared to the control, and the increased mast cell invasion was decreased by ABE‐M (Figure 4a lower panel). Moreover, ear thickness was increased by DNCB (*p* < .05, *p* < .01) (Figure 4b). However, the ABE‐M treatment group had significantly reduced epidermal and dermal thickness when compared with the AD group (Figure 4a middle panel and B). In AD conditions, predominant Th2‐type cytokines stimulate B cells to secrete IgE, which affects IgG1 and IgG2a levels (Bieber, 2008; Dokmeci & Herrick, 2008; Wood et al., 1996). To determine the inhibitory effect of ABE‐M on immunoglobulin levels in the AD model, we measured IgE, IgG1, and IgG2a levels using ELISA. DNCB‐induced mice showed an increase in IgE, IgG1, and IgG2a levels. However, the ABE‐M treatment significantly reduced the levels of these immunoglobulins (*p* < .05, *p* < .01) (Figure 4c). Especially, 200 mg/kg of ABE‐M treatment showed a superior inhibitory effect compared to DX in IgE level (*p* < .05, *p* < .01). Therefore, our results indicated that ABE‐M ameliorates AD lesions.

### 3.5 Effect of ABE‐M on the mRNA expression of chemokines and cytokines in ear tissues of DNCB‐induced BALB/c mice

**FIGURE 5:**
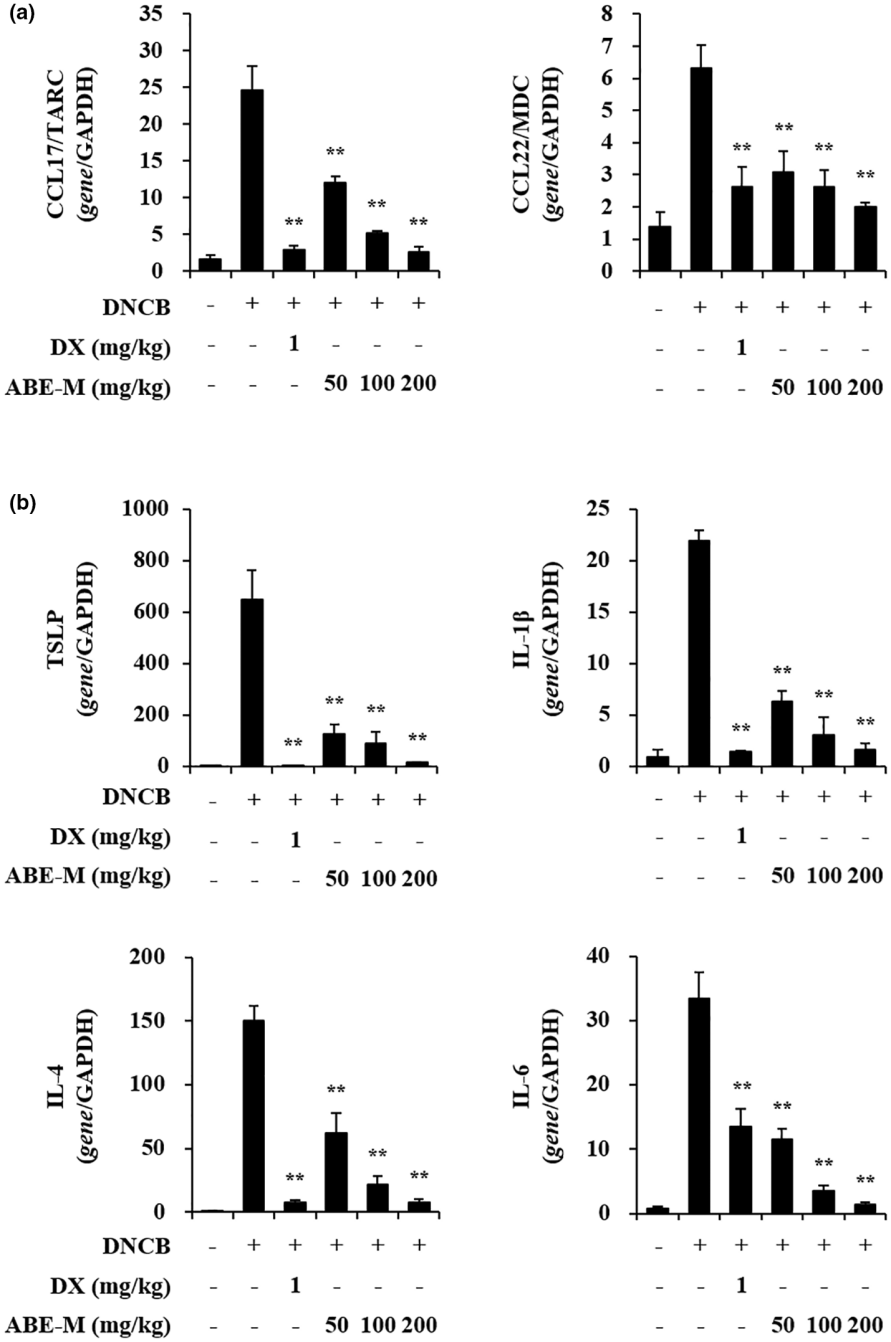
Effect of ABE‐M on pro‐inflammatory gene expression levels in DNCB‐induced AD mouse model. The expression of CCL17/TARC, CCL22/MDC, and TSLP, IL‐1β, IL‐4, IL‐6 from ear tissue was determined by real‐time PCR. *p < .05, **p < .01 compared to the DNCB group. ABE‐M, *Ampelopsis brevipedunculata* (Maxim.) Trautv. extract; AD, atopic dermatitis; CCL17/TARC, thymus and activation‐regulated chemokine; CCL22/MDC, macrophage‐derived chemokines; DNCB, 2,4‐dinitrochlorobenzene; PCR, polymerase chain reaction.

To understand the mechanism by which ABE‐M alleviates AD lesions, we investigated the expression of AD‐associated chemokines and pro‐inflammatory cytokines in ear tissues by real‐time PCR. The expression of chemokines such as CCL17 and CCL22 (*p* < .05, *p* < .01) was enhanced in the DNCB group compared to that of the control group. However, the increased gene expression was abrogated by treatment with the ABE‐M (Figure 5a). Furthermore, the pro‐inflammatory cytokines (such as TSLP, IL‐1β, IL‐4, and IL‐6) expression was significantly increased with DNCB treatment compared to the control group. However, the increased gene expression was abrogated by treatment with the ABE‐M in a dose‐dependent manner. It was found that 200 mg/kg of ABE‐M treatment decreased the gene expression to a similar degree as that of DX (*p* < .05, *p* < .01) (Figure 5b). Therefore, our results indicated that ABE‐M suppresses the gene expression of chemokines and cytokines in DNCB‐induced AD mice.

### 3.6 Effect of ABE‐M on the protein levels in ear tissues of the DNCB‐induced BALB/c mice

**FIGURE 6:**
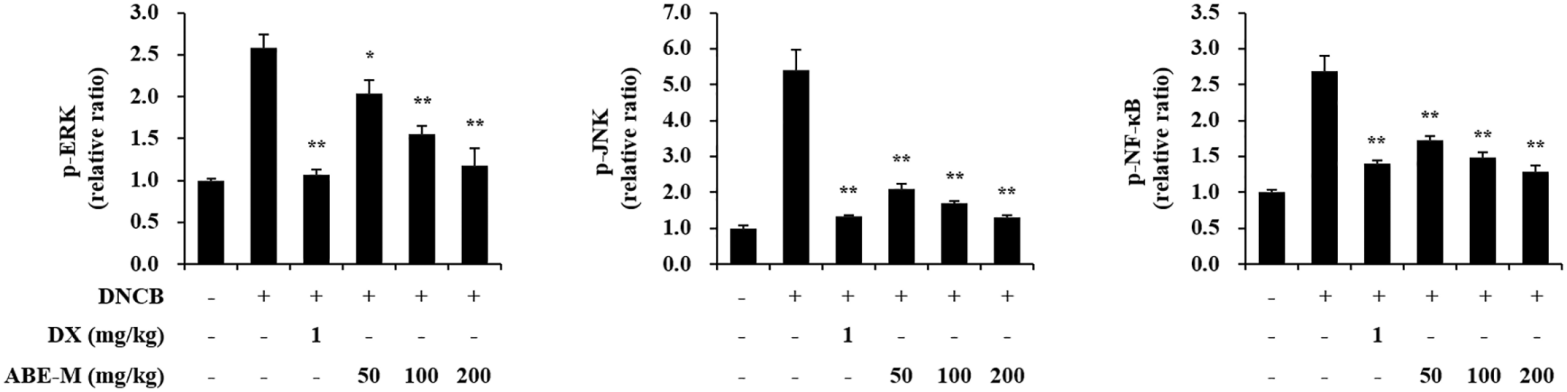
Effect of ABE‐M on signaling pathway protein levels in DNCB‐induced AD mouse model. The protein level of p‐ERK, p‐JNK, and p‐NF‐κB from ear tissue was determined by ELISA. *p < .05, **p < .01 compared to the DNCB group. ABE‐M, *Ampelopsis brevipedunculata* (Maxim.) Trautv. extract; AD, atopic dermatitis; DNCB, 2,4‐dinitrochlorobenzene; ELISA, enzyme‐linked immunosorbent assay.

The effect of ABE‐M on signaling pathways for cytokines and chemokines in ear tissues was evaluated using ELISA. After protein isolation from ear tissues, the expression levels of p‐ERK, p‐JNK, and p‐NF‐κB were measured. Protein expressions of p‐ERK and p‐JNK (*p* < .05, *p* < .01) were increased in the DNCB group compared to the control group (Figure 6). However, it was reduced with the treatment of ABE‐M. In addition, the expression of p‐NF‐κB was also inhibited (*p* < .05, *p* < .01). Therefore, it was confirmed that the elevated secretion of cytokines and chemokines by DNCB is suppressed by ABE‐M through the inhibition of these signaling pathways.

## 4 DISCUSSION

*Ampelopsis brevipedunculata* (Maxim.) Trautv. has been used widely for medicinal purposes because of its excellent antioxidant activity (Kundakovic et al., 2008) and the effect of restoring liver function (Yabe et al., 1998). In addition, previous research has reported that 95% ethanol extract of *A. brevipedunculata* (Maxim.) Trautv. is effective for treating AD (Choi et al., 2019). Our study was conducted to make an industrial product by mixing co‐solvent (dextrin) with *A. brevipedunculata* (Maxim.) Trautv., which has excellent activity. It is extracted through previously reported research. As shown in Figure S2, the yield and content of catechin and ethyl gallate, which are index components, were higher in the 50% ethanol extract than in the 95% ethanol extract. Therefore, we conducted experiments by mixing dextrin, with 50% ethanol extract. We investigated the mechanisms and effects of ABE‐M on regulating inflammatory mediators in vitro via TNF‐α/IFN‐γ stimulated HaCaT cells. First of all, when the toxicity of ABE‐M was confirmed through HaCaT cells, it seemed that there was no toxicity. CCL17/TARC and CCL22/MDC are overexpressed in keratinocytes in AD (Kwon et al., 2012). The chemokines expressed by AD upregulate Th2 cytokines. AD is an inflammatory response by Th2 cells, which is highly depend on Th2 chemokines (Akids et al., 2000; Muñoz et al., 2017). We found that the levels of inflammatory chemokines, including CCL17/TARC and CCL22/MDC, and cytokines, including IL‐1β, IL‐6, and IL‐8, increased after treatment with TNF‐α/IFN‐γ, and was inhibited when treated with ABE‐M. In addition, in the reported studies, it is known that AD is involved in MAPK and NF‐κB (Bak et al., 2023; Choi et al., 2013). Studies have shown that stimulation of TNF‐α/IFN‐γ not only activates various signaling pathways such as MAPK, STAT, and p‐NF‐κB but also increases various inflammatory chemokines/cytokines (Furukawa, Takahashi, et al., 2004). We found that ABE‐M decreased phosphorylation of ERK and JNK in a dose‐dependent manner, and the expression of NF‐κB p65 also tended to decrease. These results showed that ABE‐M co‐regulated ERK, JNK, and NF‐κB activation and significantly suppressed mRNA expression of inflammatory chemokines and cytokines. These data imply that ABE‐M has the potential to modulate inflammatory mediators.

The atopy alleviating effect of ABE‐M was verified in a DNCB‐induced AD‐like mouse model. It was visually confirmed that keratinization was alleviated in the positive control group and the ABE‐M administration group compared to the control group. In addition, in order to confirm the efficacy of ABE‐M in histological aspects, the ear tissue was stained with H&E and toluidine blue, and significant reduction in thickness and number of mast cells was confirmed in the ABE‐M group. These results demonstrated the potential of ABE‐M as an atopy improving agent. In AD, IL‐4 and IL‐5 are mainly secreted by Th2 cells, and the secreted cytokines stimulate B cells to secrete IgE, which affects IgG1 and IgG2a levels and the production of various inflammatory cytokines (Bieber, 2008; Dokmeci & Herrick, 2008; Muñoz et al., 2017). Furthermore, IgE levels are involved in the sensitization of allergens in AD (Gould et al., 2003; Werfel, 2009). As a result of examining blood immunoglobulins of AD‐like model in this study, it was confirmed that IgE, IgG1, and IgG2a were reduced by oral administration of ABE‐M. In the initiation of AD, environmental triggers promote the release of dendritic cell activation cytokines such as IL‐1β, CCL17/TARC, CCL22/MDC, and TSLP by keratinocytes (Kim et al., 2019). The TSLP‐activated dendritic cells produce CCL17/TARC and CCL22/MDC, and they promote the infiltration and differentiation of Th2 cells (Ying et al., 2005). In this study, the mRNA expression of IL‐1β, IL‐6, IL‐8, TNF‐α, and TSLP in DNCB‐induced mouse ear tissue was significantly reduced by ABE‐M. These results support that ABE‐M is a potential candidate for atopy‐improving drugs. After protein isolation from ear tissue, it was confirmed that p‐ERK, p‐JNK, and p‐NF‐κB were reduced by ABE‐M. These data imply that ABE‐M has the potential to modulate inflammatory mediators, suggesting a specific role for p‐ERK, p‐JNK, and p‐NF‐κB in alleviating AD.

Together with these reports and experimental data, we suggest that compounds containing ABE‐M synergistically alleviate atopy in vivo and in vitro. Steroid drugs commonly used to treat AD are associated with several side effects with long‐term use. Therefore, it is important to use natural resources to replace steroid drugs. Based on the findings obtained in in vitro and in vivo models of AD, ABE‐M is expected to be an effective therapeutic agent. In addition, a detailed study is needed to reveal the interaction between compounds constituting ABE‐M.

## 5 CONCLUSIONS

In conclusion, the results of our study showed that oral administration of ABE‐M ameliorates the AD‐like skin inflammation caused by DNCB. In keratinocytes, ABE‐M suppressed the expression of inflammatory cytokines and chemokines induced by TNF‐α/IFN‐γ. Similarly, the histopathological changes in DNCB‐induced mice were significantly suppressed by ABE‐M. Therefore, the immunosuppressive effect of ABE‐M shows the potential anti‐allergic activities and alleviates AD, suggesting that it can be used as a pharmacological agent.

## AUTHOR CONTRIBUTIONS

**Seon Gyeong Bak:** Conceptualization (equal); formal analysis (equal); writing – original draft (equal). **Hyung Jin Lim:** Conceptualization (equal); formal analysis (equal); writing – original draft (equal). **Yeong‐Seon Won:** Formal analysis (equal); investigation (equal). **Eun Jae Park:** Investigation (equal). **Young Hee Kim:** Investigation (equal). **Seung Woong Lee:** Data curation (equal); methodology (equal). **Je Hun Oh:** Formal analysis (equal); methodology (equal). **Ji Eun Kim:** Methodology (equal). **Min Jee Lee:** Methodology (equal). **Soyoung Lee:** Data curation (equal); investigation (equal); methodology (equal). **Mun‐Chual Rho:** Conceptualization (equal); methodology (equal). **Seung Jae Lee**: Conceptualization (equal); methodology (equal); writing – review and editing (equal).

## FUNDING INFORMATION

This work was supported by the Korea Institute of Planning and Evaluation for Technology in Food, Agriculture, Forestry (IPET) through functional food development using domestic future agricultural resources project, funded by the Ministry of Agriculture, Food and Rural Affairs (MAFRA) (821024–03), and the KRIBB Research Initiative Program (KGM5242322). This research was funded by grants from the National Research Council of Science & Technology (NST) funded by the Korean government (MIST), grant numbers CRC21022‐100.

## CONFLICT OF INTEREST STATEMENT

The authors declare that they have no competing interests.

## ETHICS STATEMENT

Animal care and treatment protocols were conducted according to the guidelines established by the Public Health Service Policy on the Humane Care and Use of Laboratory Animals and were approved by the Institutional Animal Care and Use Committee of the Korea Research Institute of Bioscience and Biotechnology (approval number: KRIBB‐AEC‐21259).

## CONSENT FOR PUBLICATION

Not applicable.

## Supporting information


Figure S1.
Figure S2.

## Data Availability

The datasets generated and analyzed during the current study are available from the corresponding author upon reasonable request.
