# Supplementary material for: Effect of Ampelopsis brevipedunculata (Maxim.) Trautv extract on a model of atopic dermatitis in HaCaT cells and mice
Source: Food Sci Nutr. 2023 Aug 17;11(10):6616–25. doi: 10.1002/fsn3.3610 (PMC10563673; doi:10.1002/fsn3.3610)
Supplement: Supplementary file 1 — Figure S1. Figure S2. [file FSN3-11-6616-s001.docx]

**Supplementary Material**

**Effect of *Ampelopsis brevipedunculata* (Maxim.) Trautv extract on a model of atopic dermatitis in HaCaT cells and mice**

Seon Gyeong Bak^1, #^, Hyung Jin Lim^1, #^, Yeong-Seon Won^1^, Eun Jae Park^1^, Young Hee Kim^2^, Seung Woong Lee^1^, Je Hun Oh^3^, Ji Eun Kim^3^, Min Jee Lee^3^, Soyoung Lee^1^, Seung Jae Lee^1,4, *^, Mun Chual Rho^1,*^

^1^ *Functional Biomaterial Research Center, Korea Research Institute of Bioscience and Biotechnology (KRIBB), Jeongeup 56212, Korea*

^2^ *Division of Biotechnology and Advanced Institute of Environment and Bioscience, College of Environmental and Bioresource Sciences, Jeonbuk National University, Iksan, 54596, Korea*

^3^ *Ju Yeong NS Co., Ltd., Seoul, 05854, Korea*

^4^ *Applied Biological Engineering, KRIBB School of Biotechnology, University of Science and Technology, Daejeon 34113, Korea*

^#^ These authors equally contributed to this study.

^*^Correspondence and requests for materials should be addressed to S.-J.L and M.C.R (email: seung99@kribb.re.kr & rho-m@kribb.re.kr)


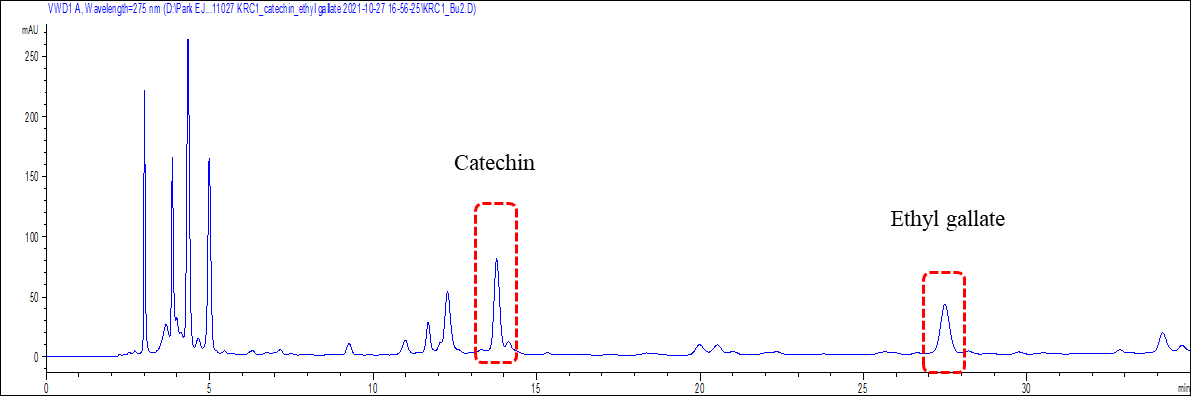


**Supplementary figure 1.** HPLC chromatogram of ABE.


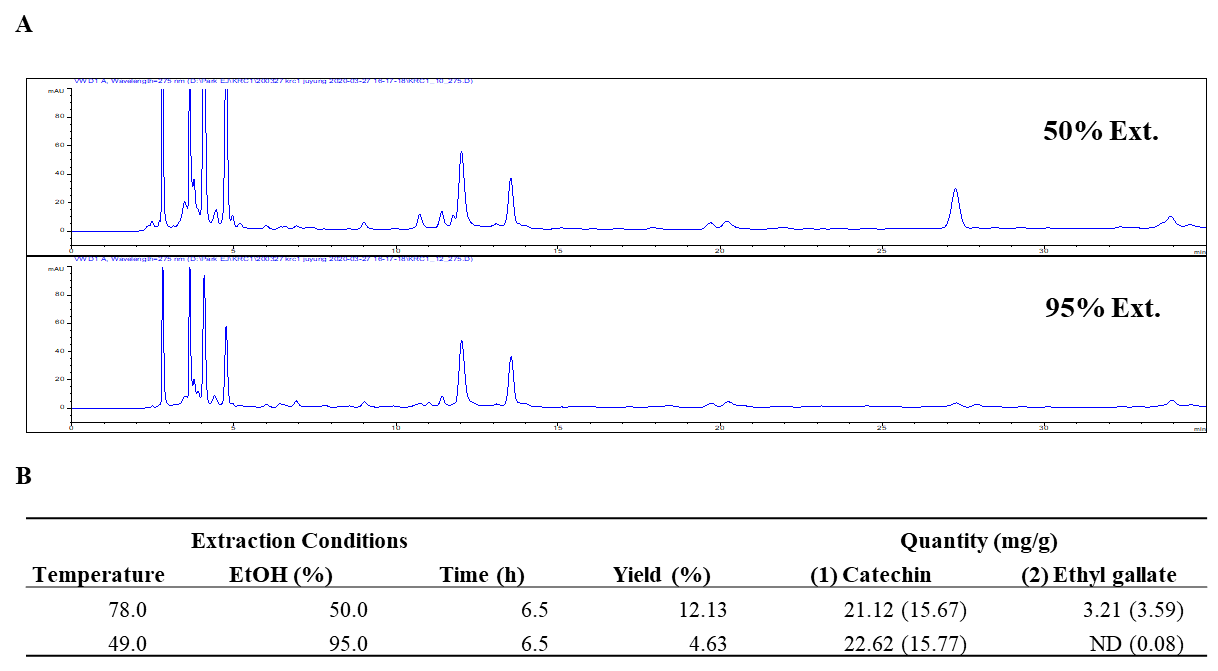


**Supplementary figure 2.** Componential analysis of ABE 50% and 95% extraction.
